# Supplementary figures and images for: Plant pathogens convergently evolved to counteract redundant nodes of an NLR immune receptor network
Source: PLoS Biol. 2021 Aug 23;19(8):e3001136. doi: 10.1371/journal.pbio.3001136 (PMC8412950; doi:10.1371/journal.pbio.3001136)

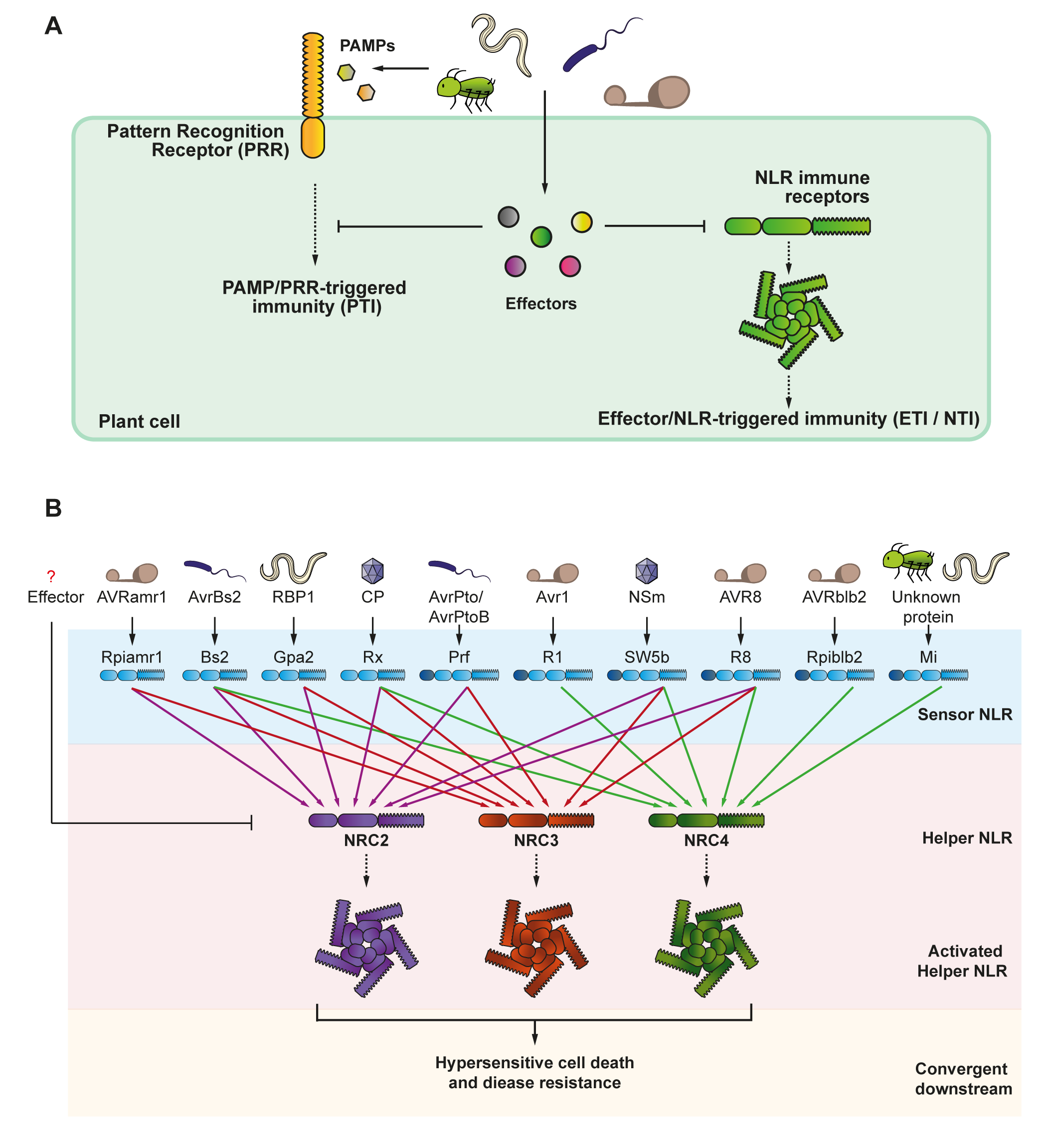

Supplement: S1 Fig — (A) ETI/NTI suppressing effectors counteract the activation of NLR immunity elicited by other effectors with an AVR activity (AVR effectors). The majority of effectors studied to date are known to suppress PTI by acting on various host targets involved in PTI signalling. However, we know little about the mechanisms of NTI suppressing effectors and how these effectors counteract NLR receptor functions and/or resistosome formation (B) The NRC network of Solanaceae. Helper NLRs (NRC2, NRC3, and NRC4) function redundantly with a series of R genes that confer resistance against multiple pathogens. These R genes encode NLR sensors that have specialised in detecting effectors from pathogens as diverse as oomycetes, bacteria, nematodes, viruses, and aphids. Many of the NRC-dependent R genes are agronomically important. Sensor NLRs (blue) with N-terminal extensions possess an additional domain at the N-terminus, represented in dark blue. The extent to which pathogen effectors have evolved to target the NRC helpers are currently unknown. Figure is adapted from [24,55]. AVR, avirulence; ETI/NTI, effector/NLR-triggered immunity; NLR, nucleotide-binding domain and leucine-rich repeat; NRC, NLR required for cell death; PAMP, pathogen-associated molecular pattern; PRR, pattern recognition receptor; PTI, PRR-triggered immunity. (TIF) [file pbio.3001136.s001.tif]

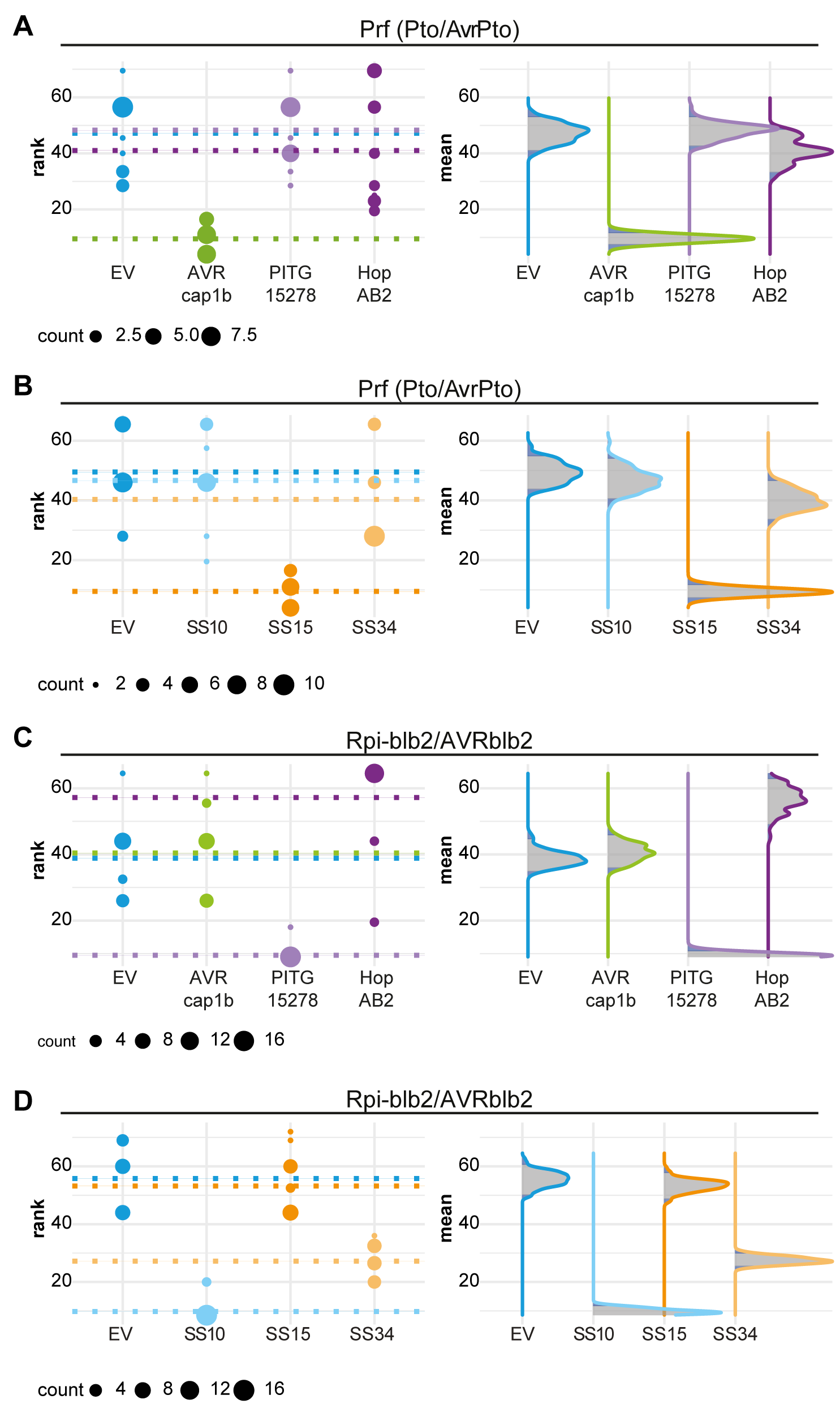

Supplement: S2 Fig — Statistical analysis was conducted using besthr R library (MacLean, 2019) (S4 Data). (A–D) Each panel corresponds to results from Pto/AvrPto or Rpi-blb2/AVRblb2 (labelled above), coexpressed with either EV (dark blue), AVRcap1b (green), HopAB2 (dark purple), PITG-15278 (light purple), SS10 (light blue), SS15 (dark orange), or SS34 (light orange), where EV was used as a negative control, labelled below plots. The left panel represents the ranked data (dots) and their corresponding means (dashed lines), with the size of each dot proportional to the number of observations for each specific value (count key below each panel). We performed bootstrap resampling tests using a lower significance cutoff of 0.025 and an upper cutoff of 0.975. Mean ranks of test samples falling outside of these cutoffs in the control samples bootstrap population were considered significant. The panels on the right shows the distribution of 1,000 bootstrap sample rank means, where the blue areas under the curve illustrate the 0.025 and 0.975 percentiles of the distribution. EV, empty vector. (TIF) [file pbio.3001136.s002.tif]

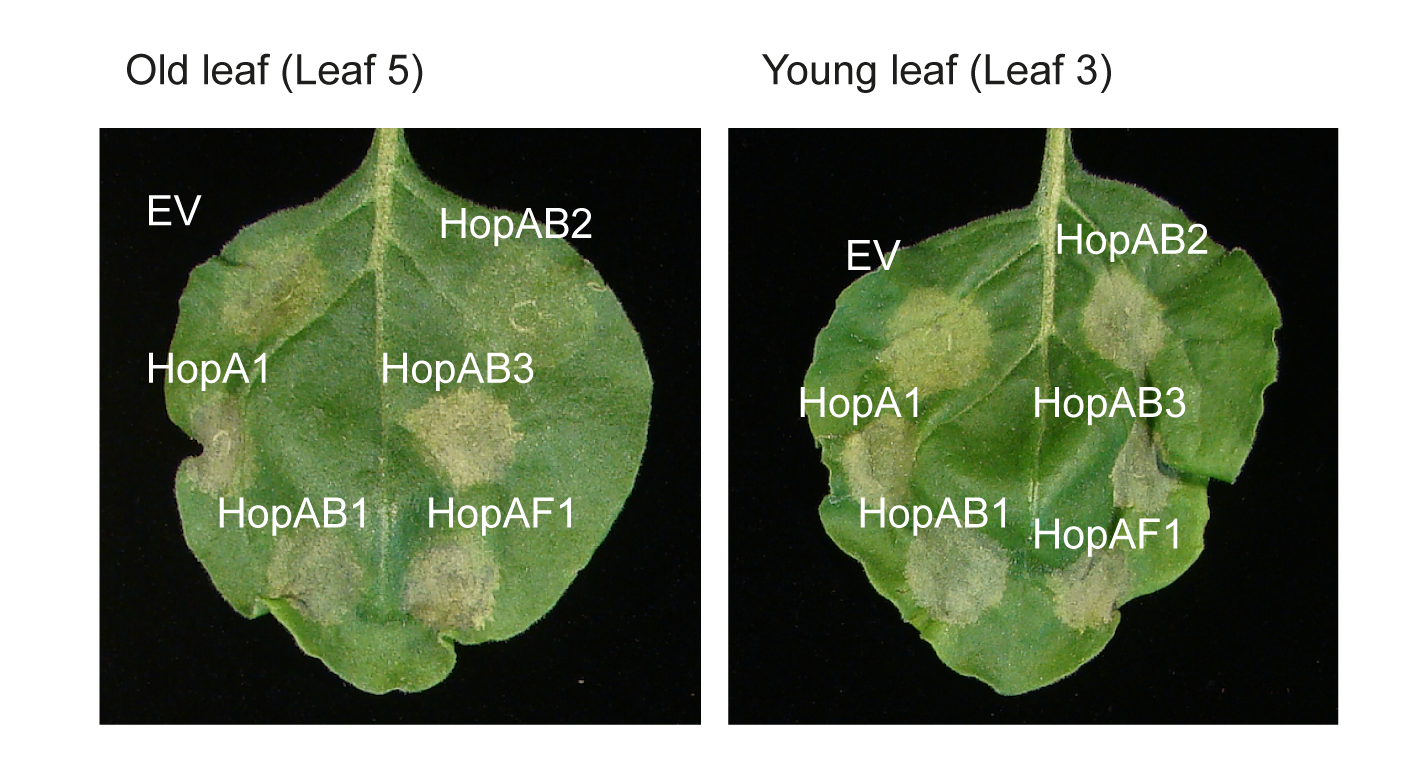

Supplement: S3 Fig — P. syringae effectors HopA1, HopAB1, HopAB2, HopAB3, and HopAF1 were transiently coexpressed with Pto/AvrPto in N. benthamiana leaves. The leaves were photographed 5 days after infiltration. HopAB2 suppression of Pto/AvrPto in N. benthamiana were evident on leaf 5 (old leaf, left), but not on leaf 3 (young leaf, right) as defined by [39]. Photographs are representative of 3 technical repeats. EV, empty vector. (TIF) [file pbio.3001136.s003.tif]

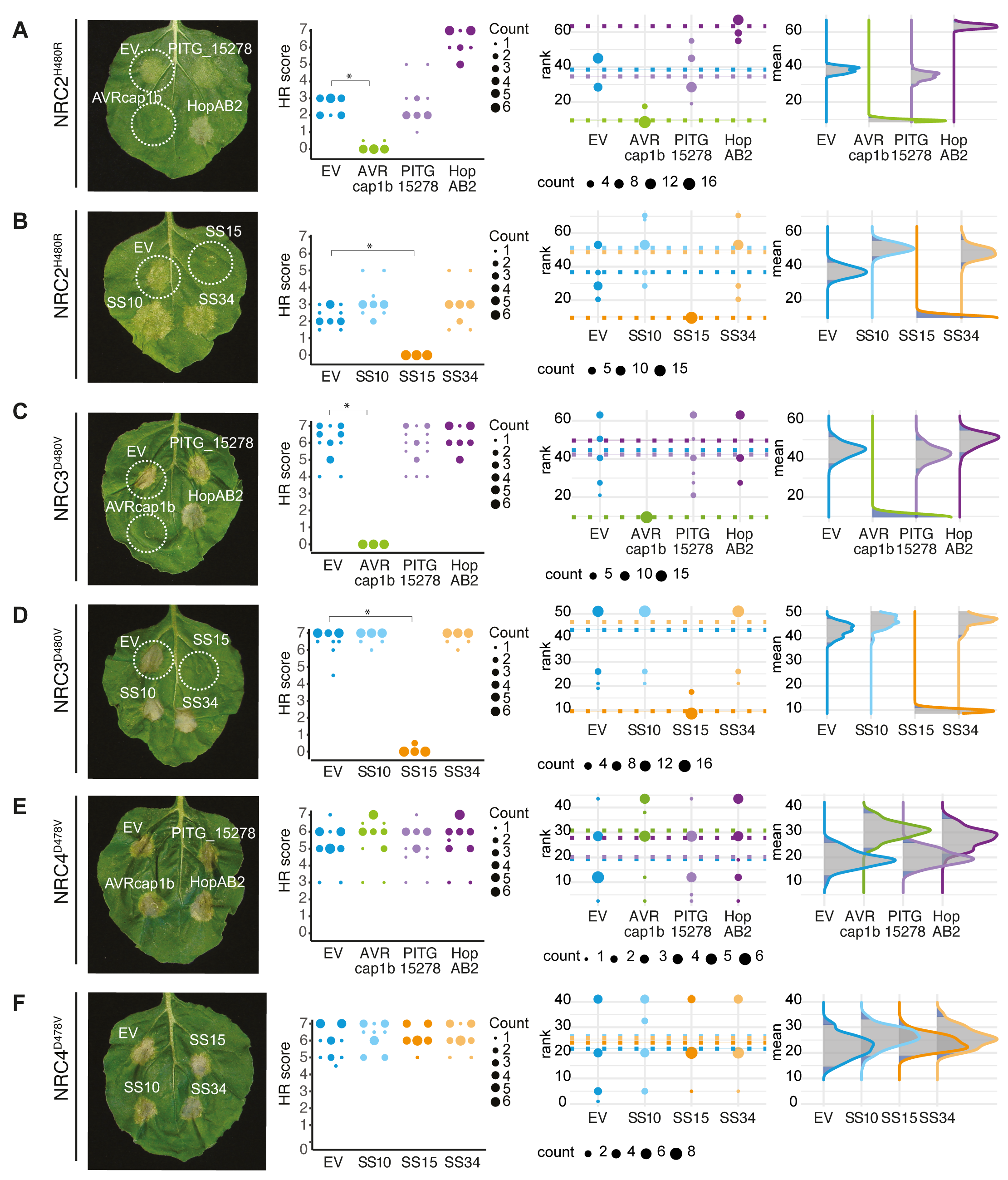

Supplement: S4 Fig — (A–F) Leaf panels: Photo of representative N. benthamiana leaves showing HR after coexpression of autoimmune NRC2H480R, NRC3D480V, and NRC4D478V mutants, indicated to the left of leaf panels, with either EV, AVRcap1b, PITG_15278, HopAB2, SS10, SS15, or SS34 (labelled on leaf) (S4 Data). Middle panels: HR was scored 5 days post-agroinfiltration, using a modified 0–7 scale (Segretin and colleagues) and photographed at 5 days after agroinfiltration. HR results are presented as dot plots, where the size of each dot is proportional to the number of samples with the same score (count) within each biological replicate. Each effector is represented by a different dot colour (see key right side of plot). The experiment was independently repeated 3 times each with 6 technical replicates. The columns of each tested effector correspond to results from different biological replicates. Right panels: Statistical analysis was conducted using besthr R library (MacLean, 2019). The dots represent the ranked data and their corresponding means (dashed lines), with the size of each dot proportional to the number of observations for each specific value (count key below each panel). The panels on the right show the distribution of 1,000 bootstrap sample rank means, where the blue areas under the curve illustrates the 0.025 and 0.975 percentiles of the distribution. A difference is considered significant if the ranked mean for a given condition falls within or beyond the blue percentile of the mean distribution of the EV control. Significant differences between the conditions are indicated with an asterisk (*). EV, empty vector; HR, hypersensitive response. (TIF) [file pbio.3001136.s004.tif]

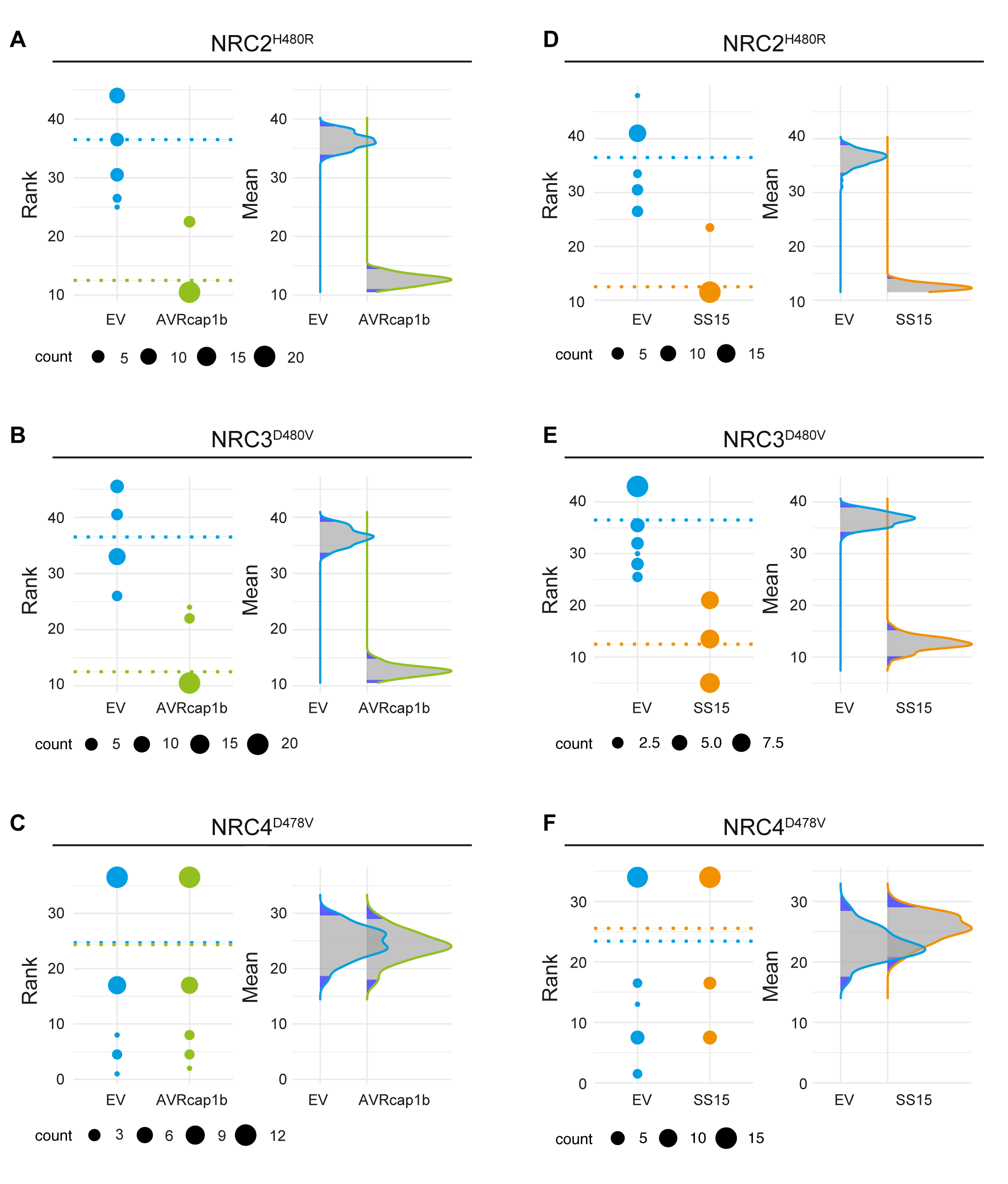

Supplement: S5 Fig — Statistical analysis was conducted using besthr R library (MacLean, 2019) (S5 Data). Each panel corresponds to results from (A, D) NRC2H480R, (B, E) NRC3D480V, or (C, F) NRC4D478V (labelled above), coexpressed with either EV (blue), AVRcap1b (green), or SS15 (orange), where EV was used as a negative control (labelled under plots). The left panel represents the ranked data (dots) and their corresponding means (dashed lines), with the size of each dot proportional to the number of observations for each specific value (count key below each panel). The panels on the right show the distribution of 1,000 bootstrap sample rank means, where the blue areas under the curve illustrate the 0.025 and 0.975 percentiles of the distribution. A difference is considered significant if the ranked mean for a given condition falls within or beyond the blue percentile of the mean distribution of the EV control. EV, empty vector. (TIF) [file pbio.3001136.s005.tif]

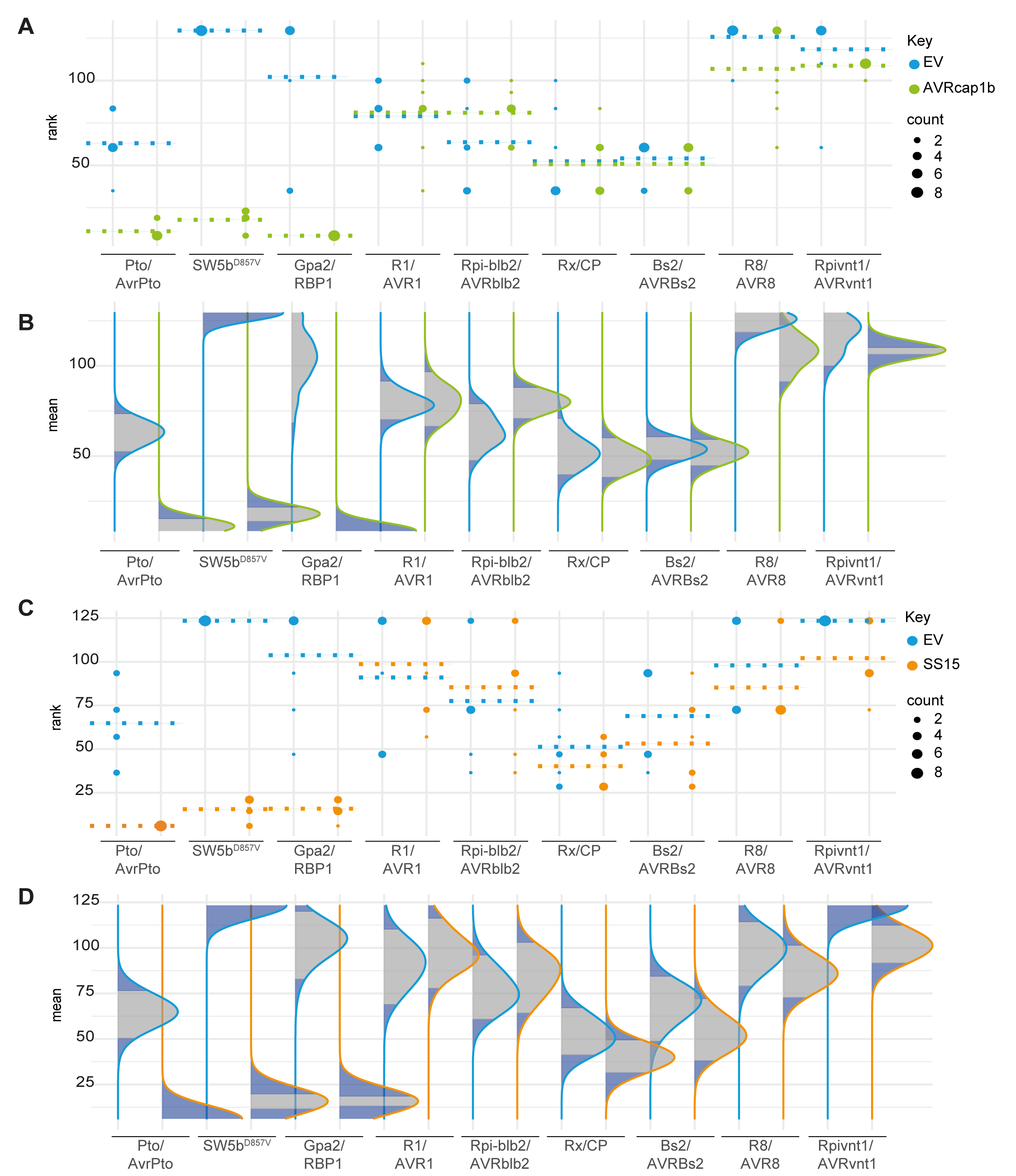

Supplement: S6 Fig — Statistical analysis was conducted using besthr R library (MacLean, 2019) (S6 Data). (A–D) Within each panel results from a range of different sensor NLRs (labelled below) were coexpressed with either EV (blue), AVRcap1b (green), or SS15 (orange), where EV was used as a negative control (see key on right hand side). Panels (A) and (C) represent the ranked data (dots) and their corresponding means (dashed lines), with the size of each dot proportional to the number of observations for each specific value (refer to count key, on left hand side). Panels (B) and (D) show the distribution of 1,000 bootstrap sample rank means, where the blue areas under the curve illustrate the 0.025 and 0.975 percentiles of the distribution. A difference is considered significant if the ranked mean for a given condition falls within or beyond the blue percentile of the mean distribution of EV control for each treatment. EV, empty vector; NLR, nucleotide-binding domain and leucine-rich repeat. (TIF) [file pbio.3001136.s006.tif]

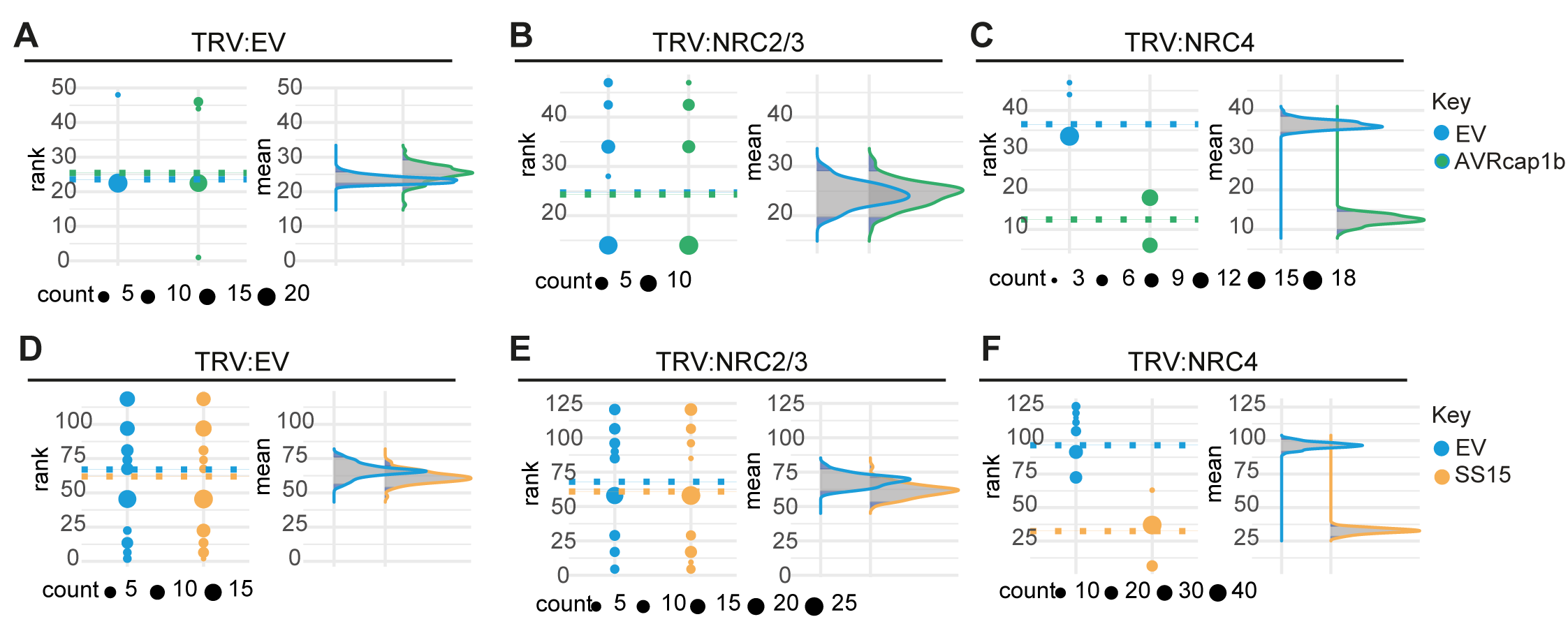

Supplement: S7 Fig — Statistical analysis was conducted using besthr R library (MacLean, 2019) (S7 Data). (A–F) Each panel represents a different silencing treatment (labelled above). Left plot represents the ranked data (dots) and their corresponding means (dashed lines), with the size of each dot proportional to the number of observations for each specific value (refer to count key below each individual panel). Right plot shows the distribution of 1,000 bootstrap sample rank means, where the blue areas under the curve illustrate the 0.025 and 0.975 percentiles of the distribution. A difference is considered significant if the ranked mean for a given condition falls within or beyond the blue percentile of the mean distribution of EV control. EV, empty vector. (TIF) [file pbio.3001136.s007.tif]

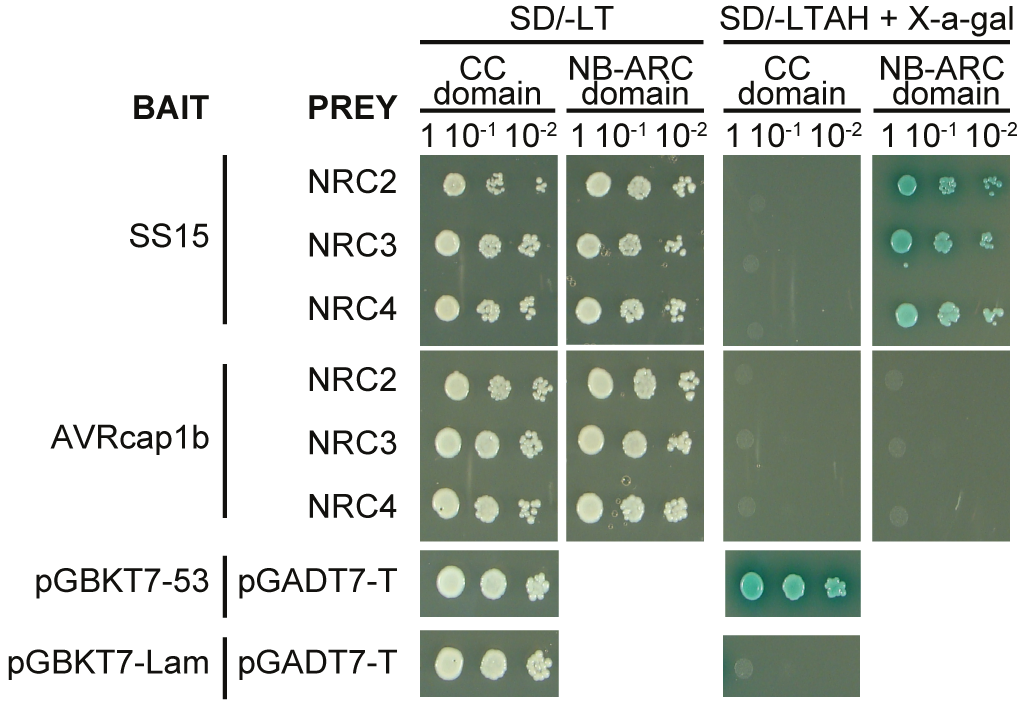

Supplement: S8 Fig — Y2H assay of SS15 and AVRcap1b with CC and NB-ARC domains of NRC2, NRC3, and NRC4. Control plates for yeast growth are on the left (SD-Leu-Trp, ST0048, Takara Bio, USA), with quadruple dropout media supplemented with X-a-gal on the right (SD-Leu-Trp-Ade-His, ST0054, Takara Bio, USA). The commercial yeast constructs were used as positive (pGBKT7-53/pGADT7-T) and negative (pGBKT7-Lam/pGADT7-T) controls. Growth and development of blue colouration in the selection plate are both indicative of protein–protein interaction. SS15 and AVRcap1b were fused to the GAL4 DNA-binding domain (Bait), and the truncated NRCs were fused to the GAL4-activator domain (Prey). Each experiment was repeated 3 times with similar results. CC, coiled-coil; NB-ARC, nucleotide-binding domain shared with APAF-1, various R-proteins, and CED-4; NRC, NLR required for cell death; Y2H, yeast two-hybrid. (TIF) [file pbio.3001136.s008.tif]

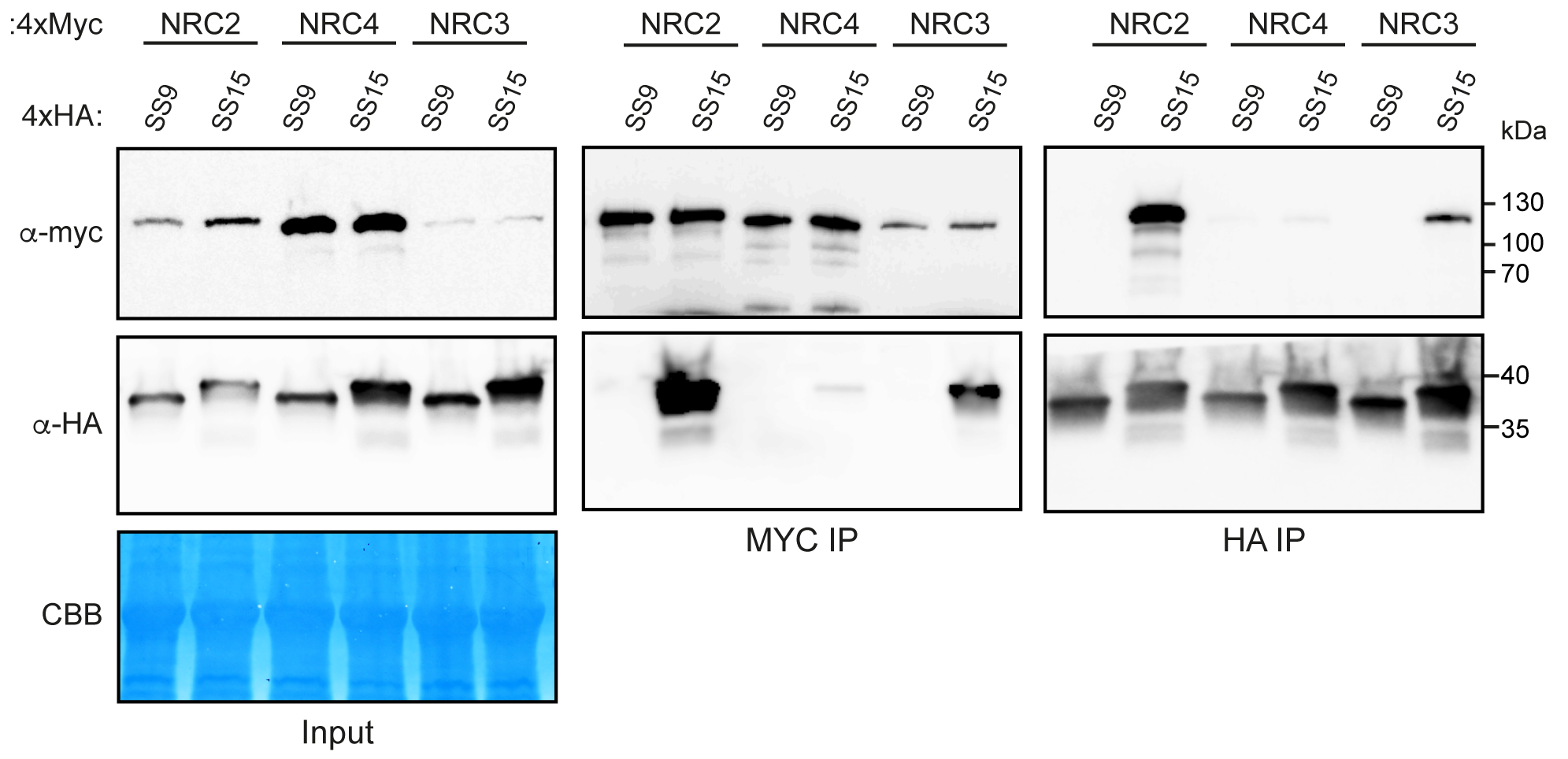

Supplement: S9 Fig — N-terminally 4xHA-tagged SS15 (SS15) and SS9 (SS9) were individually transiently coexpressed with NRC proteins fused to a C-terminal 4xMyc tag. SS9 was used as a negative control. IP were performed with agarose beads conjugated to either anti-Myc (MYC IP) or anti-HA (HA IP) antibodies. Total protein extracts were immunoblotted with appropriate antisera labelled on the left. Approximate molecular weights (kDa) of the proteins are shown on the right. Rubisco loading controls were conducted using CBB staining. This experiment is a representative of 2 independent replicates. CBB, Coomassie brilliant blue; coIP, co-immunoprecipitation; IP, immunoprecipitation. (TIF) [file pbio.3001136.s009.tif]

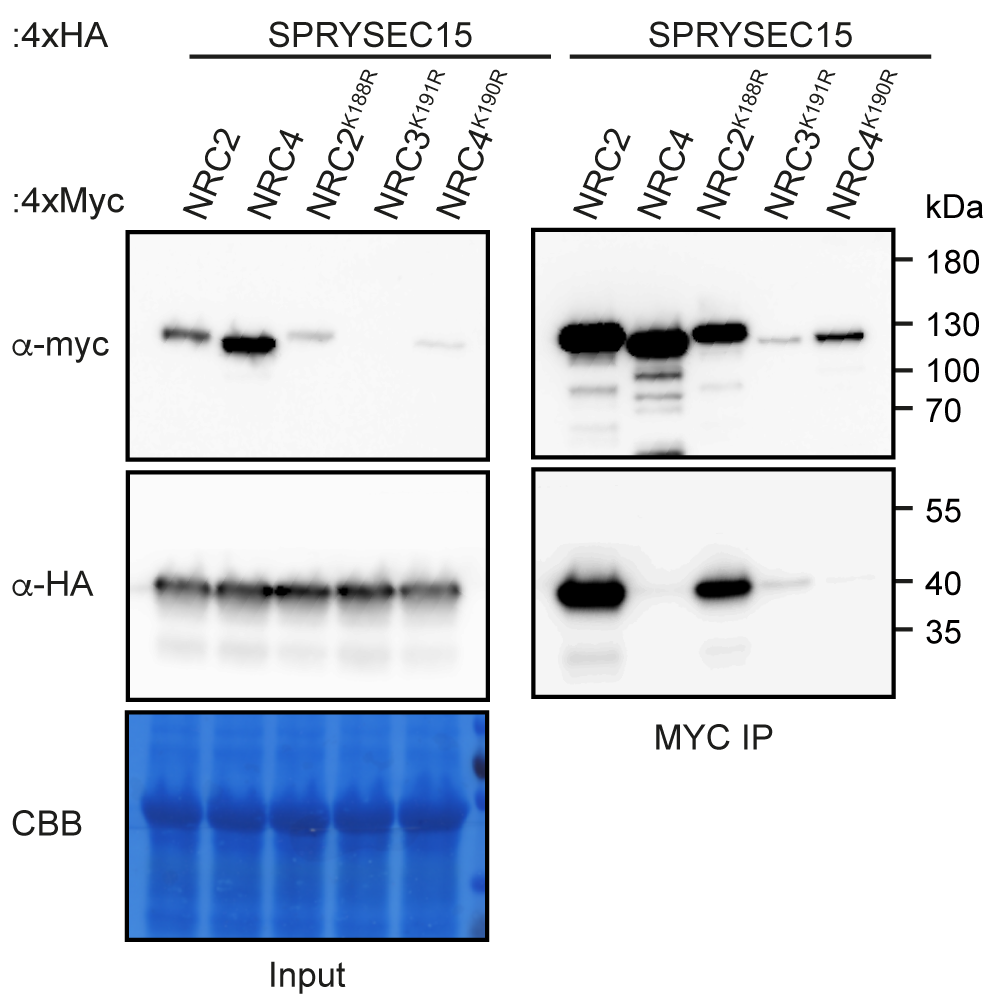

Supplement: S10 Fig — (A) N-terminally 4xHA-tagged SS15 was transiently coexpressed in N. benthamiana with C-terminally 4xMyc-tagged NRC2, NRC4, NRC2K188R, NRC3K191R, and NRC4K190R. IP was performed with agarose beads conjugated to anti-Myc (Myc IP) antibodies. Total protein extracts (Input) and proteins obtained by coIP were immunoblotted with appropriate antisera labelled on the right. Approximate molecular weights (kDa) of the proteins are shown on the right. Rubisco loading controls were conducted using CBB staining. This experiment is representative of 3 independent replicates. CBB, Coomassie brilliant blue; coIP, co-immunoprecipitation; IP, immunoprecipitation. (TIF) [file pbio.3001136.s010.tif]

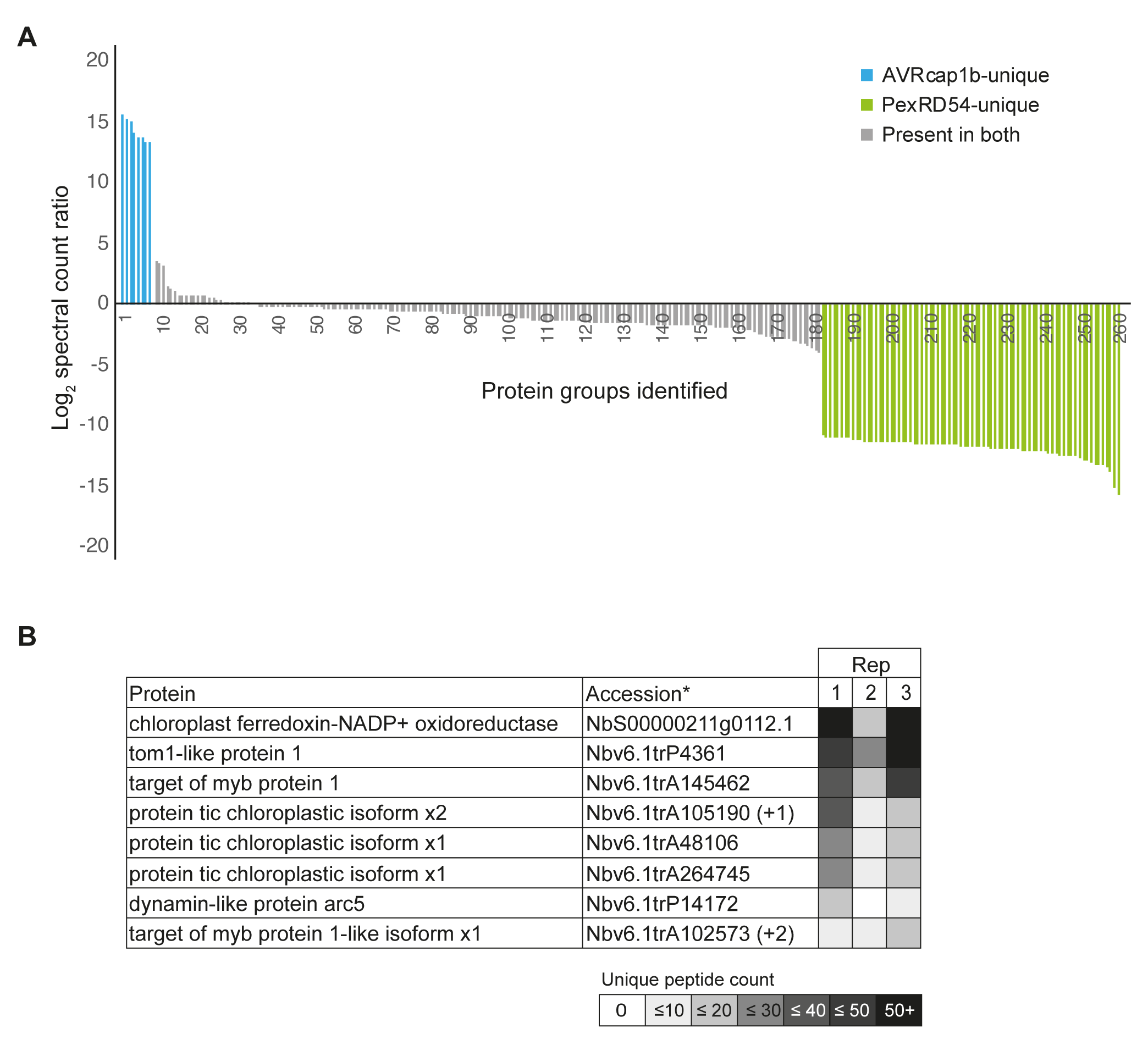

Supplement: S11 Fig — (A) Immune affinity enriched protein abundance plot generated based on proteins groups identified for AVRcap1b and PexRD54. Protein abundances were calculated as the total mean spectral counts identified with each effector across 3 replicates. The Log2 of the ratio of mean counts observed for AVRcap1b divided by the mean counts observed for PexRD54 was calculated. All 261 proteins identified were then sorted from highest to lowest ratio and plotted in this order (see S14 Table for details). Proteins with the highest ratio correspond to the most likely AVRcap1b (blue) specific interactors. Proteins most likely to correspond to PexRD54 are labelled in green. Proteins in grey were identified in both effectors. (B) Eight putative unique AVRcap1b proteins (based on previously mentioned ratio of mean spectral counts) identified in IP-MS experiment. Unique spectral counts obtained in each of the 3 independent replicates with AVRcap1b are included on the right-hand side. IP-MS, immunoprecipitation–mass spetrometry. (TIF) [file pbio.3001136.s011.tif]

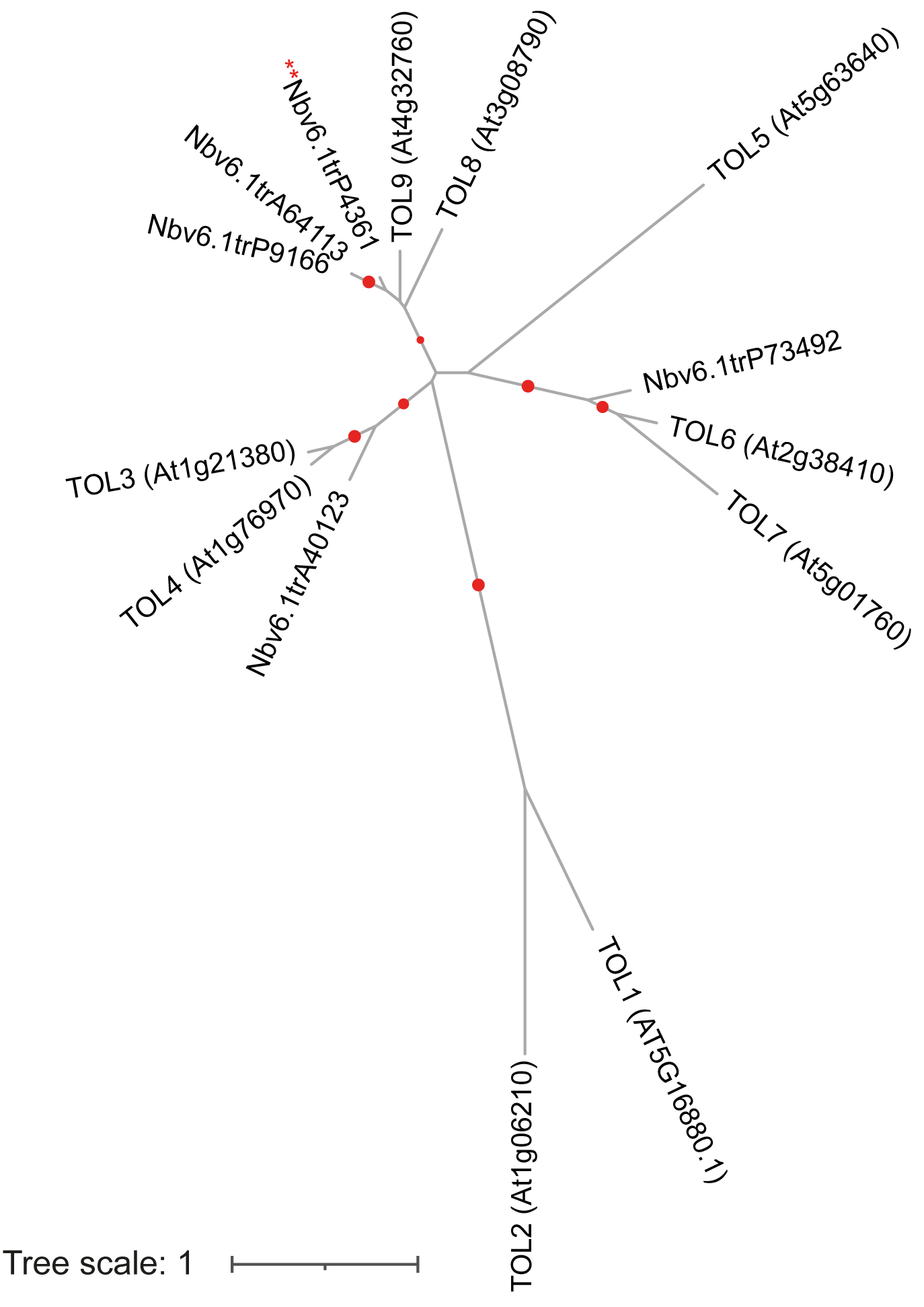

Supplement: S12 Fig — Protein sequences were aligned using Clustal Omega. The ENTH and GAT domains were used for further analysis. The phylogenetic tree was constructed in MEGAX using the JTT substitution model and 1,000 bootstrap iterations. Branches with bootstrap support higher than 80 are indicated with red dots. NbTOL9a is indicated by 2 red asterisks. The scale bar indicates the evolutionary distance in amino acid substitutions per site. JTT, Jones–Taylor–Thornton; TOL, Target of Myb 1-like protein. (TIF) [file pbio.3001136.s012.tif]

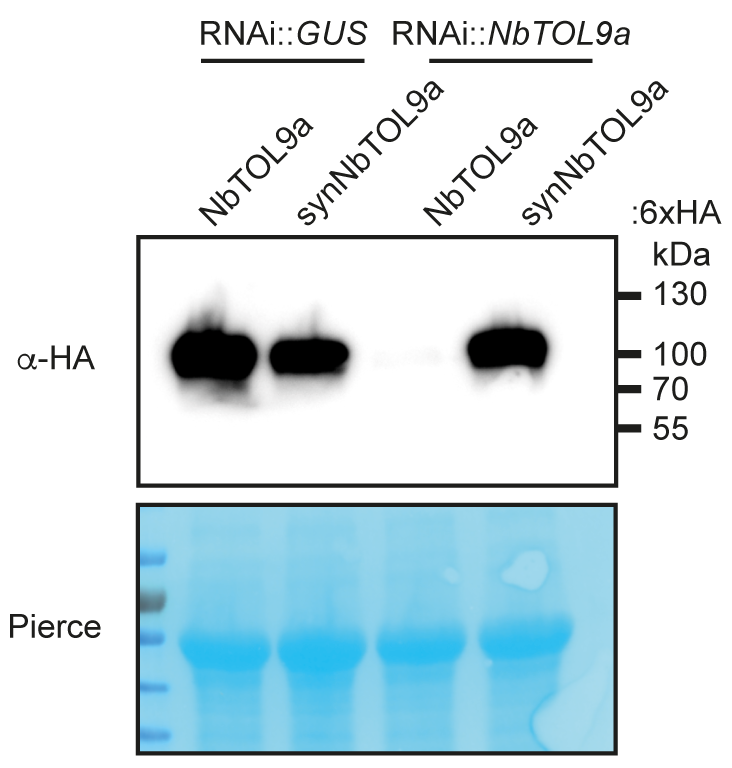

Supplement: S13 Fig — WT NbTOL9a (NbTOL9a::6HA) and the synthetic version (synNbTOL9a::6xHA) were transiently coexpressed with RNAi::GUS or RNAi:NbTOL9a. synNbTOL9a::6xHA was used as a control that is not targeted for knockdown by the RNAi::NbTOL9a. Total protein extracts were immunoblotted with HA antiserum. Approximate molecular weights (kDa) of the proteins are shown on the right. Rubisco loading controls were conducted using Pierce staining. WT, wild type. (TIF) [file pbio.3001136.s013.tif]

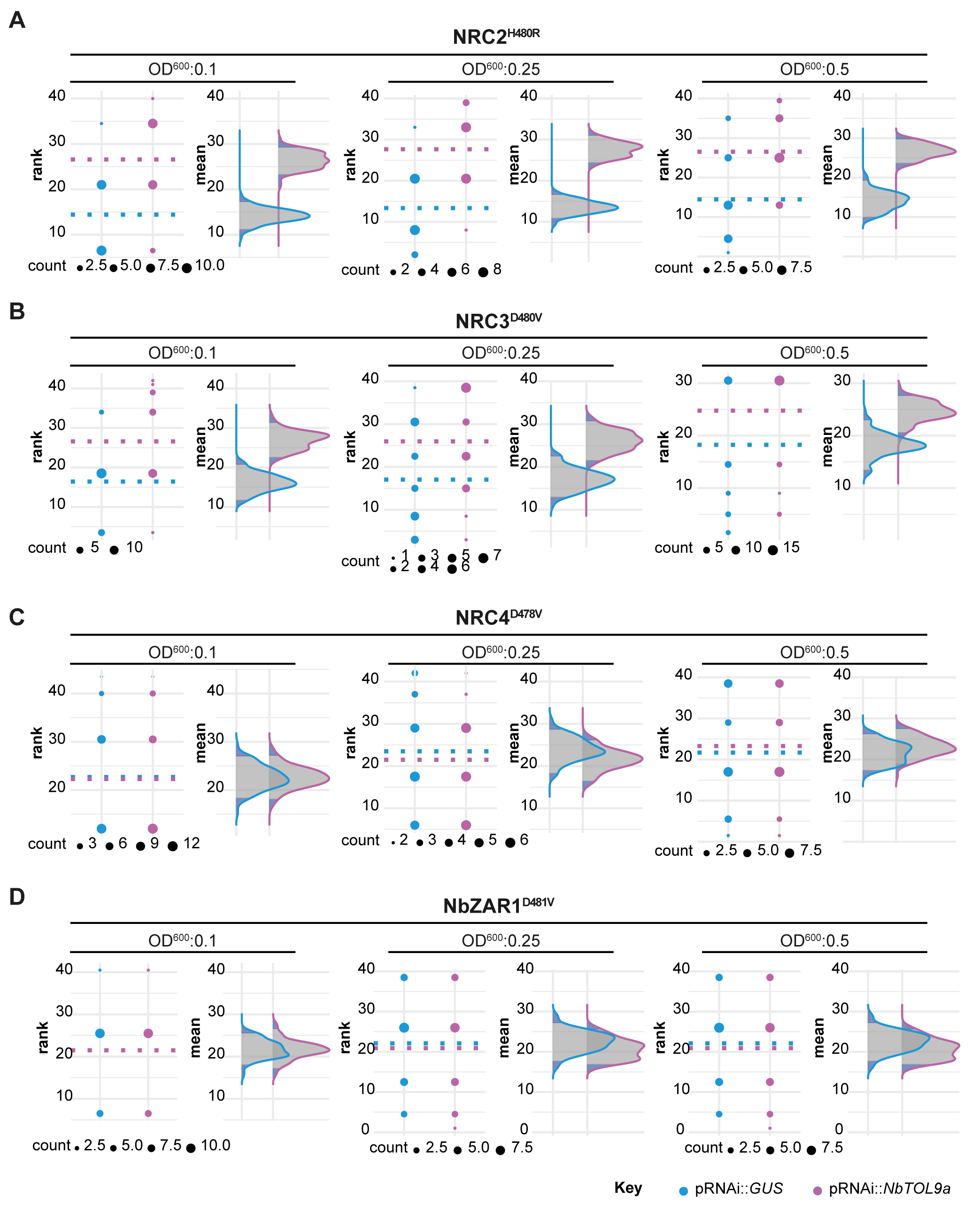

Supplement: S14 Fig — Statistical analysis was conducted using besthr R library (MacLean, 2019) (S10 Data). Each panel represents increasing concertation of A. tumefaciens expressing NRC3D480V and NRC4D478V (OD600 = 0.1, 0.25, or 0.5). (A) NRC2H480R, (B) NRC3D480V, (C) NRC4D478V, and (D) ZAR1D481V. The left most panel represents the ranked data (dots) and their corresponding means (dashed lines), with the size of each dot proportional to the number of observations for each specific value (refer to count legend bottom of figure). The right panel shows the distribution of 1,000 bootstrap sample rank means, where the blue areas under the curve illustrate the 0.025 and 0.975 percentiles of the distribution. A difference is considered significant if the ranked mean for RNAi::NbTOL9a (purple) falls within or beyond the blue percentile of the mean distribution of RNAi::GUS (blue) for each treatment combination. (TIF) [file pbio.3001136.s014.tif]

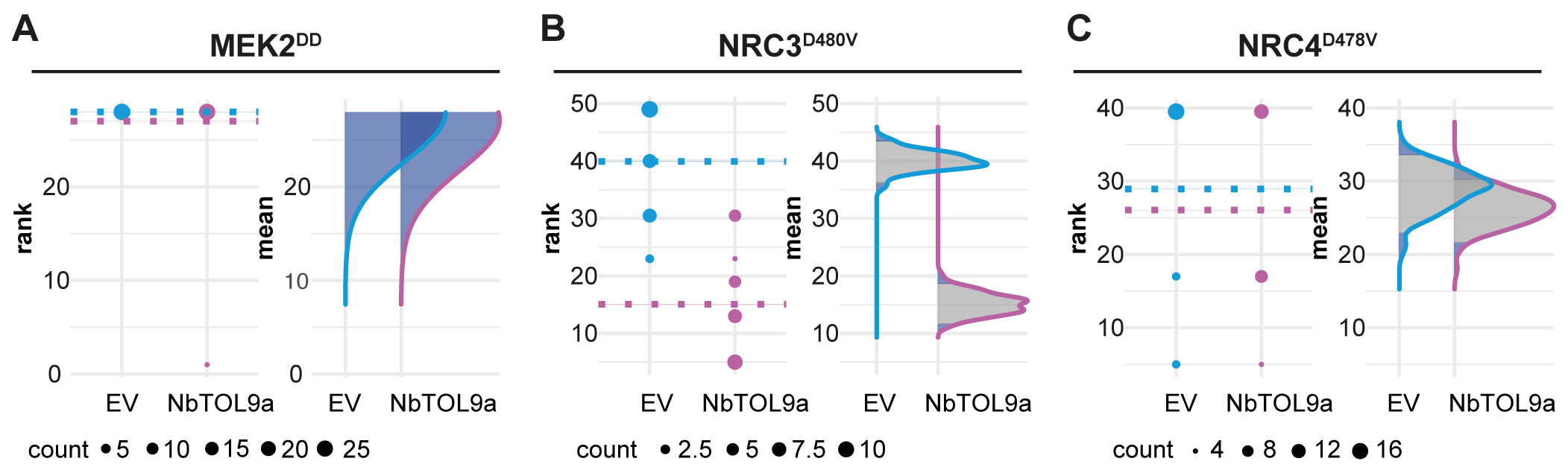

Supplement: S15 Fig — Statistical analysis was conducted using besthr R library (MacLean, 2019) (S11 Data). For (A) MEK2DD, (B) NRC3D480V, and (C) NRC4D478V, the left most panel represents the ranked data (dots) and their corresponding means (dashed lines), with the size of each dot proportional to the number of observations for each specific value (refer to count legend bottom of figure). The right panel shows the distribution of 1,000 bootstrap sample rank means, where the blue areas under the curve illustrate the 0.025 and 0.975 percentiles of the distribution. A difference is considered significant if the ranked mean for AVRcap1b (purple) falls within or beyond the blue percentile of the mean distribution of EV control (blue) for each treatment (MEK2DD, NRC3D480V, and NRC4D478V), in the presence of either EV or NbTOL9a. EV, empty vector; HR, hypersensitive response. (TIF) [file pbio.3001136.s015.tif]

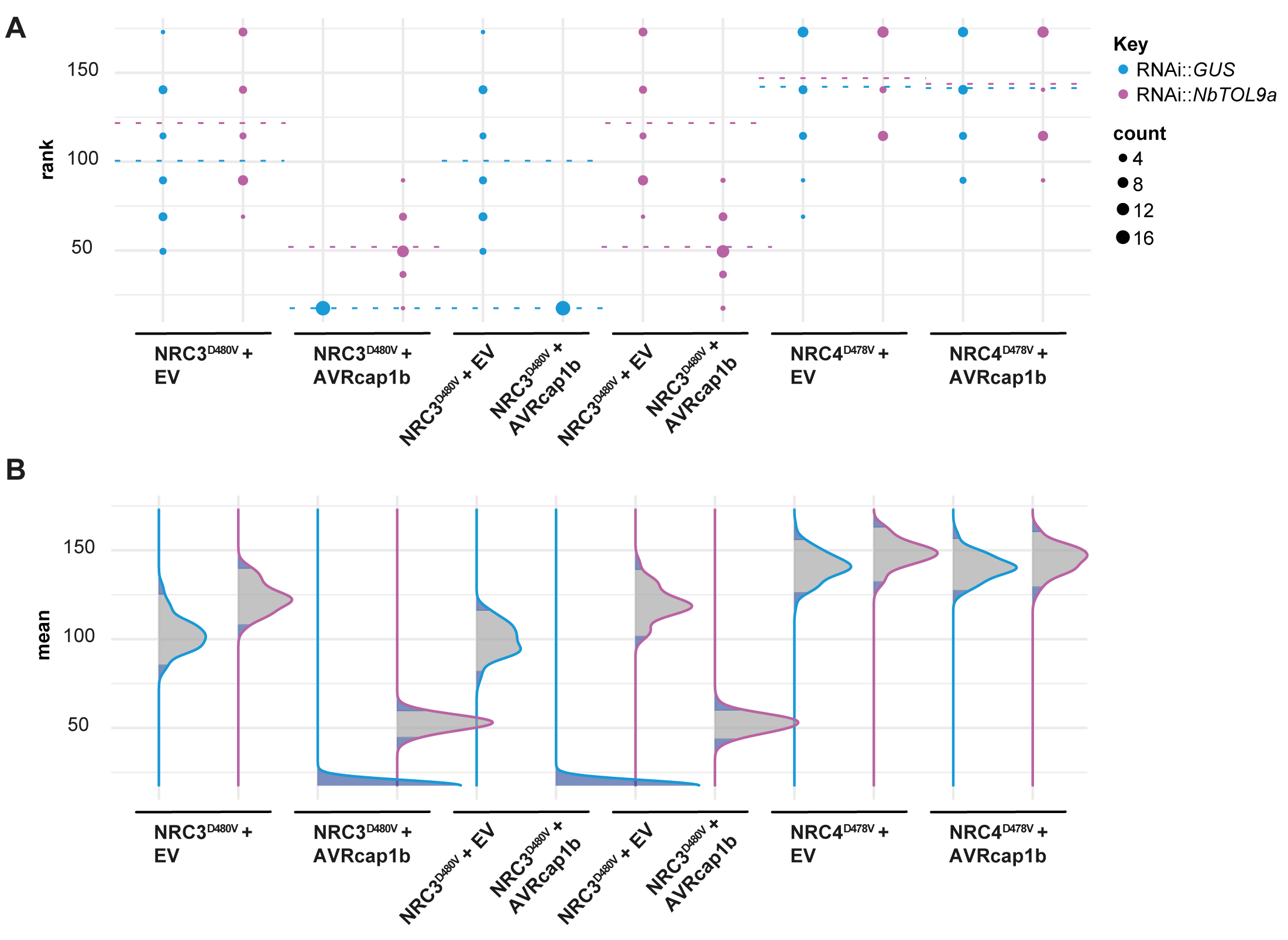

Supplement: S16 Fig — Statistical analysis was conducted using besthr R library (MacLean, 2019) (S12 Data). Autoactive NRC3 (NRC3D480V) was coexpressed with either EV (negative control) or AVRcap1b (labelled on bottom of plot), with RNAi::GUS (blue) or RNAi::NbTOL9a (purple) silencing treatments. (A) represents the ranked data (dots) and their corresponding means (dashed lines), with the size of each dot proportional to the number of observations for each specific value (refer to count key, on right hand side). (B) shows the distribution of 1,000 bootstrap sample rank means, where the blue areas under the curve illustrate the 0.025 and 0.975 percentiles of the distribution. A difference is considered significant if the ranked mean for a given condition falls within or beyond the blue percentile of the mean distribution of the corresponding RNAi::GUS controls within each given treatment (NRC3D480V + EV, NRC3D480V + AVRcap1b, NRC4D478V + EV, and NRC4D478V + AVRcap1b). EV, empty vector. (TIF) [file pbio.3001136.s016.tif]
