# Supplementary material for: Plant pathogens convergently evolved to counteract redundant nodes of an NLR immune receptor network
Source: PLoS Biol. 2021 Aug 23;19(8):e3001136. doi: 10.1371/journal.pbio.3001136 (PMC8412950; doi:10.1371/journal.pbio.3001136)
Supplement: Attachment — Submitted filename: PBIOLOGY-D-21-00375R1_-_Response_to_reviewers_Final.docx [file pbio.3001136.s045.docx]

**PBIOLOGY-D-21-00375R1 Response letter**

We thank the Editors and the Reviewers of PLOS Biology for their valuable time, insights, and critical observations on our manuscript. We aimed to accommodate the reviewers' concerns as much as possible and have compiled the following point by point responses to the concerns posed. Line numbers mentioned in our responses refer to the resubmitted materials.

# **EDITOR COMMENTS**

## Editor comment 1 (key point)

In particular, both reviewers think that you should discuss the different results you find in the Y2H and IP assays.

## Author’s response

This is indeed an interesting point. We mention that NLRs are rarely recovered in Y2H screens (Lines 363 – 365), which may explain why NRC2 was not recovered in these assays. However, it is also possible that NRC2 or its subdomains may not accumulate well in the yeast strain used in the blind Y2H screen performed by Hybrigenics Services, thereby preventing identification of positive hits. We have added some thoughts to the revised manuscript (lines 367 – 369).

Additionally, although association between SS15 and NRC4 was detected in Y2H, the association between these two proteins is not detectable (Figure 6) or very weak (mentioned in lines 398 – 401, also see Figure S9) in coIP experiments. It is possible that the physiological conditions in plants do not favour the association between SS15 and NRC4, consistent with the results that SS15 does not suppress NRC4 in the cell death assays. We have included some thoughts in the revised manuscript (lines 665 – 669).

## Editor comment 2 (key point)

## 2a

Reviewer #1 thinks that a greater diversity of NLRs should be tested if they want to claim specificity to this node in the network, including non-NRC-dependent NLRs, says that it’s not clear why NRC2 was omitted, but in his opinion, the importance of the paper does not depend on this.

## Author’s response

We have conducted further RNAi knockdown experiments and updated the accompanying figures (Figure 10 and Fig S14) to include NRC2 as well as the NRC-independent NLR, NbZAR1. Using constitutively active forms of NRC2 (NRC2^H480R^) and NbZAR1 (NbZAR1^D481V^), we show that NRC2^H480R^, but not NbZAR1^D481V^, is affected by NbTOL9a silencing. Together with our previous data, these results support the hypothesis that NbTOL9a-mediated negative regulation of cell death is specific to NRC2 and NRC3 and not NRC4 or NbZAR1, a non-NRC dependent NLR (see lines 515 – 522).

In subsequent experiments we focused on NRC3^D480V^ as it gave a stronger autoimmune response compared to NRC2^H480R^, and provides a more robust readout for cell death-based assays. We have now explained this rationale in text to clarify our omission of NRC2 (line 540 – 543).

## 2b

You have to clarify the biological basis to choose only some effectors, in particular only one family of the nematode effectors

## Author’s response

The aim of our study was to determine whether effectors evolved to target the NRC immune receptor network. To do this, we tested effectors from an array of different Solanaceae parasites. For nematodes, we focused on SPRYSEC effectors because they arguably form the largest and most well-known effector family, with many reportedly involved in immunity suppression (e.g. Ali et al 2015; Postma et al 2012; Mei et al 2018). Of course, we would have ideally tested all effectors secreted by these parasites, but that would be beyond the scope of this paper. We have added some text to address this in the revised manuscript (lines 169 – 171).

## 2c

Reviewer #1 says that your data only shows that NbTOL9a is required for NRC3 and is not entirely convinced that the cyst nematodes and P. infestans effectors target the same node.

## Author’s response

We have added a section explaining our rationale for focusing on NRC3 in subsequent AVRcap1b experiments and have modified the text accordingly (also see comment 2a).

To address the reviewers concern in regard to the effectors targeting the same node, we modified the text as recommended by the reviewer (lines 634 – 637).

## Editor comment 3 (key point)

Reviewer #2 wants you to do an experiment to resolve whether TOL9a interacts directly with NRCs to suppress AVRcap1b mediated cell death.

## Author’s response

This is indeed a key experiment. To address this, we tested association between NbTOL9a and NRCs by *in planta* coIP. We show that NbTOL9a does not associate with NRCS and this lack of association is unaffected by the presence/absence of AVRcap1b (Lines 590 – 607, 741 – 742, Figure 13). We have included this in the revised manuscript.

# **Reviewer #1 (Comments for the Author):**

## Reviewer 1 comment 1 (key point)

Very convincingly, these two were confirmed to suppress a range of NRC2/3 dependent, but not NRC4 dependent, NLRs. The authors then identify interactors of these two effectors in an unbiased Y2H screen. One of which identified several clones corresponding to NRC3 and 4, but not 2. It is extremely encouraging to see these data, although why NRC2 was missing and NRC4 present (and yet in IP we get 2 and 3) could be discussed further.

## Author’s response

Please see points discussed in Editor’s comment 1.

## Reviewer 1 comment 2 (key point)

Importantly, it is then shown that a TOL is a negative regulator of auto active NRC3 but not NRC4 nor MEK2 - with the conclusion that it is specific to this node in the network (I think a greater diversity of NLRs should be tested if this claim is wanted to be made, including non-NRC-dependent NLRs). It’s not clear why NRC2 was omitted, but again, in my opinion, the importance of the paper does not depend on this. I recommend the manuscript is accepted following minor revisions to the text to either additionally clarify, or rephrase, some of the points raised below.

## Author’s response

We have provided additional experiments that further support our claim that NbTOL9a negative regulation is specific to NRC2 and NRC3 and not other NLRs (see response to editor’s comment 2a). We have also added text to the revised manuscript to address the rationale behind focusing on NRC3 in subsequent experiments (see response to editor’s comment 2a).

## Reviewer 1 comment 3 (key point)

## It seems there was a drastic pre-screen of effectors before being tested here (at least for the nematode effectors, only a single family was chosen - omitting most of the diversity of known effectors, including many families involved in immunity suppression) - the biological basis for this choice should be made clear.

## Author’s response

We have provided a rationale for our selection of effectors (see response to editor’s comment 2b).

## Reviewer 1 comment 4 (key point)

## It is rather neat that RBP1:GPA2 is suppressed by SS15, because RBP1 is itself a SPRYSEC. Paints a nice picture of one SPRYSEC coming to the rescue of another. Also, rather intriguingly, SS15 seems to be recognised in tabacum - outside the scope of this paper but would be fascinating to know what by, and whether NRC dependent.

## Author’s response

We agree this is quite interesting, and further highlights the complex coevolutionary dynamics that exist between effectors and NLRs. It would indeed be interesting to identify the gene involved in recognising SS15 in *N. tabacum*, although, as the reviewer mentions, this is out of the scope of this paper. We have however, added text mentioning this in the updated version of the manuscript (Lines 750 – 761).

Reviewer 1 comment 5 (key point)

Curious that SS10 and 34 also suppress NRC4-mediated Rpi-blb2/AVRblb2 - but apparently not directly (or at least not the auto active versions). The suggestion is made that they must target either the sensor or block the interaction between the sensor and the helper. Struggling a little to understand why they may block the sensor, if that sensor does not recognise a cognate nematode effector - perhaps it points to something in between? Would have been nice to see whether they also suppress R1/AVR1, or what their interactors are, but this is not necessary for this manuscript - perhaps a point for discussion.

## Author’s response

This is indeed an interesting finding. The Rpi-blb2 family is a large NLR clade with features of significant recent expansion. It is possible that close orthologs of Rpi-blb2 from other *Solanum* species mediate immunity against cyst nematodes, thus, *G.* *rostochiensis* evolved two effectors (SS10 and SS34) to suppress these Rpi-blb2 orthologs rather than Rpi-blb2 per se. However, it is also possible that these effectors block the sensor-helper interaction (Rpiblb2-NRC4). We plan to test SS10 and SS34 suppression of R1 (and other NLR) – mediated cell death in the future. We have included some thoughts in the discussion (lines 776 – 779).

## Reviewer 1 comment 6 (key point)

## The claim that AVRcap1b and SS15 suppress Rx-mediated cell death only in the absence of NRC4 seems to be supported by the data, but it does differ from the literature. This should at least be noted, as it is with HopAB2.

## Moffett, Peter, et al. "Analysis of *Globodera rostochiensis* effectors reveals conserved functions of SPRYSEC proteins in suppressing and eliciting plant immune responses." Frontiers in plant science 6 (2015): 623.

## Author’s response

## This data does indeed contrast to those published by Moffett et al, who showed that SS15 partially suppresses Rx – mediated cell death. While we cannot exclude the possibility that the growth conditions or genetic background of wild type *N. benthamiana* lead to these differences in suppression activity, in all our experiments involving Rx, we were only able to show suppression of Rx-mediated cell death in the absence of NRC4 (Figure 3 and Figure 4). We agree that this should be noted in text. We have included this in the revised manuscript (lines 257 – 258). Reviewer 1 comment 7 (key point)

"We found that SS15 directly binds the NB-ARC domain of NRC2 and NRC3, while AVRcap1b associates with NbTOL9a and requires this host protein to fully suppress NRC2 and NRC3."
Data only shown that NbTOL9a is required for NRC3?

## Author’s response

We have conducted experiments to show that NbTOL9a negatively regulates both NRC2 and NRC3, but not NRC4 or NbZAR1. We have updated the manuscript to address this (see response to editor comment 2a). However, as the reviewer points out, we do not have data to specifically show that AVRcap1b requires NbTOL9a to fully suppress NRC2. We have modified the text accordingly (lines 574 – 576, 648 – 649).

## Reviewer 1 comment 8 (key point)

## "We conclude that cyst nematodes and P. infestans convergently evolved effectors that target the same key nodes of the NRC network to suppress host immune signalling." I am not entirely convinced that they target the same node, if AVRcap1b is doing something downstream? By the same logic, it could be argued that AVRcap1b also targets the sensor nodes. Without knowing that TOLs are *exclusive* to this node (showing not involved in NRC4 is not sufficient), it seems a little far reaching. Even then, isn't it just the next node in a network? Addressing this could be as simply as "at or downstream of the same node", or "signalling from the same node".

## Author’s response

We had touched upon this in the initial version of the manuscript but have tried to make it clearer in the updated version. We modified the text as recommended by the reviewer (see editor comment 2c).

Reviewer 1 comment 9 (key point)

Several comments with the text of Figure 13:
"*P. infestans* and *G. rostochiensis*, two evolutionary unrelated pathogens, have evolved effectors that suppress signaling mediated by the NRC network."
I know the intended meaning, but they are not unrelated. Notwithstanding that, I agree with the premise of this sentence as opposed to the above.

## Author’s response

We have re-worded this (Lines 642):

"*P. infestans* and *G. rostochiensis*, two evolutionary distinct pathogens, have evolved effectors that suppress signaling mediated by the NRC network."

Reviewer 1 comment 10 (key point)

"The cyst nematode effector, SS15, can suppress both inactive and activated NRC2 and NRC3 by binding the NB-ARC domain."
I don't think there is any data to show it can suppress the inactive forms, and thinking about it I'm not sure what that means?

## Author’s response

Thank you for pointing this out. We have modified the text to make it clearer (lines 645 – 648).

Reviewer 1 comment 11 (key point)

"The P. infestans effector, AVRcap1b, can suppress the function of NRC2 and NRC3 by associating with the ESCRT-related protein NbTOL9a."
Only data shown for NRC3 not NRC2?

## Author’s response

We have provided additional data showing that NbTOL9a is also a negative regulator of NRC2 (see response to Editor comment 2a). However, as the reviewer pointed out, we do not have data to show AVRcap1b suppression of NRC2 occurs through association with NbTOL9a, we have therefore modified the text accordingly (Line 648 – 649).

Reviewer 1 comment 12 (minor point)

Figure 2:
What is the R domain of AVRcap1b and the yellow unlabelled domain of SS15?
Can infiltrated areas be indicated on c as in Figure 1c?
I know it read, "The details of statistical analysis are presented in Figure S4." But I really think at least the name of the test and the cutoff value used should be listed in the main figure and the text. This goes for all figures.

## Author’s response

Thank you for bringing this to our attention. We have now clarified this in the figure legend and updated the figure (lines 228). We have also highlighted infiltrated areas, as per the reviewer’s suggestion. All statistical test information has also been included in all main figures.

Reviewer 1 comment 13 (minor point)

Figure 5:
Its not clear from the diagram how extreme resistance and susceptibility are differentiated.

## Author’s response

This is indeed confusing. Since we have used Rx transgenic *N. benthamiana* plants, all tested plants are resistant (or display extreme resistance to PVX). Only when Rx resistance is compromised do we see trailing necrosis, as is the case with NRC4 and NRC2/3/4 silenced lines. To simplify this, we removed the susceptible example from diagram (see figure 5).

Reviewer 1 comment 14 (minor point)

SPRYSEC is already an acronym. Consider not using an acronym of an acronym and keeping SPRYSEC.

## Author’s response

We thank the reviewer for this comment. We decided to use SS instead of SPRYSEC to keep the effector name brief. We have clearly defined this in the manuscript (lines 142) and used SS consistently throughout.

Reviewer 1 comment 15 (minor point)

While they are variously used in the literature, I am not sure I agree that parasite is an umbrella term for pests and pathogens - authors may wish to rephrase.

## Author’s response

We thank the reviewer for this comment. Our use of parasite is according to the definition by Méthot and Alizon:

“Parasite: any organism that decreases the fitness of its host by infecting it. This ecological definition includes both micro-parasites (e.g., viruses and bacteria) and macro-parasites (e.g., worms).”

This thus encompasses oomycetes, nematodes, bacteria and aphids.

Méthot and Alizon (2014) What is a pathogen? Towards a process view of host-parasite interactions. Virulence 5(8) 775 – 785.  doi: [10.4161/21505594.2014.960726](https://dx.doi.org/10.4161%2F21505594.2014.960726)

Reviewer 1 comment 16 (minor point)

"Parasites as diverse as bacteria, oomycetes, nematodes and aphids turned out to be much more sophisticated manipulators of their host plants than anticipated." 
Were they ever considered unsophisticated? - consider rephrasing.

## Author’s response

We agree that plant parasites are sophisticated manipulators of their host plants. They are, however, far more sophisticated than we initially anticipated. We have clarified this in text (line 57).

Reviewer 1 comment 17 (minor point)

"Interestingly, even though AVRcap1b is a robust NTI suppressor, it can activate immunity on accessions of *Solanum capsicibaccatum* carrying the NLR disease resistance gene Rpi-cap1- hence the moniker AVR (33, 75). The fact that AVRcap1b can act as both a trigger and suppressor of NLR immunity further highlights the complex coevolutionary dynamics that exist between effectors and NLRs and the need for studies that take into account these intricate epistatic interactions."
Same looks to be true for SS15 in *N. tabacum*.

## Author’s response

## This is a very interesting point that indeed was not touched upon in the discussion. We have now added text to address this in the updated version of the manuscript (Lines 750 – 761, also see reviewer 1 comment 4).

# **Reviewer #2 (Comments for the Author):**

## To identify proteins that interact with these two effectors they performed Y2H screen. The co-IP data presented shows that SS15 effector interacts with NRC2 and NRC3 but not with NRC4.

Reviewer 2 comment 1 (key point)

-    In the Y2H experiment, the authors recovered NRC3 and NRC4 with SS15. However, in the co-IP experiments it only interacted with NRC3. In co-IP the authors show that SS15 also interacts with NRC2 but this was not detected in Y2H screen. Some discussion should be included with respect to the different assays showing different results. The data shown indicate that SS15 interacts directly with the NB-ARC domain of NRC4 in vitro. Furthermore, it interacts with both activated and inactivated NRC2 in planta.

## Author’s response

Please see points discussed in Editor’s comment 1.

Reviewer 2 comment 2 (key point)

To understand how AVRcap1b suppresses cell death, the authors performed pull down experiments and identified TOL family protein. Using co-IP, transient overexpression and silencing experiments, the data shown indicate that AVRcap1b may use TOL9a host factor to suppress NRC-mediated cell death.

-    Does TOL9a interact directly with NRCs to suppress AVRcap1b mediated cell death? 

## Author’s response

We have now included a new results section (section “NbTOL9a does not associate with NRCs *in planta*”) and accompanying figure (Figure 13) in which we used *in planta* co-IP to address this question (see Editor comment 3). Our results indicate that TOL9a does not associate with NRC2, NRC3 or NRC4 *in planta* (Lines 590 – 607, 741 – 742).
